# Time to rethink prevention of neonatal group B streptococcal disease

**DOI:** 10.1007/s00281-026-01080-1

**Published:** 2026-09-16

**Authors:** Jana Lucia Teuscher, Florens Lohrmann, Egbert Herting, Fumi Sugihara, Philipp Henneke, Christoph Härtel

**Affiliations:** 1 Campus Lübeck, Clinic for Pediatric and Adolescent Medicine, University Hospital Schleswig-Holstein, Lübeck, Germany; 2 Department of Pediatrics, University Hospital Würzburg, Josef-Schneider-Straße 2, 97080 Würzburg, Germany; 3 Center for Pediatrics and Adolescent Medicine, University Hospital Freiburg, Freiburg, Germany; 4 Institute for Infection Prevention and Control, University Hospital Freiburg, Freiburg, Germany; 5 Specialist Network Infectious Diseases, Netzwerk Universitätsmedizin, Berlin, Germany

**Keywords:** Group B streptococcus, GBS, Neonate, Immunity, Microbiome, Intrapartum prophylaxis, Antibiotics

## Abstract

Group B Streptococci (GBS) are both natural colonizers and important invasive pathogens in newborns. The introduction of intrapartum antibiotic prophylaxis has led to a significant decrease in the incidence of early onset sepsis. In contrast, late onset sepsis, which accounts for up to 50% of cases, has not declined. IAP impacts microbiome development during infancy, specifically it reduces the abundance of *Bifidobacterium* and *Bacteroides* spp., which might be associated with long-term health outcomes such as asthma development and obesity. This review highlights the challenges and risks associated with GBS colonization in neonates, including the role of virulence factors and specific contribution of host immunity. Bacterial pathogenicity factors involved in adhesion and colonization, e.g. GBS pilus islands, barrier disruption, e.g. pore-forming toxin ß-hemolysin, or evasion of neonatal immune defense mediated by GBS capsular polysaccharide and membrane glycolipids are important targets to suppress the translocation of GBS and progression to invasive disease. In line with this, metabolic programming of neonatal immune cells, e.g. glycolytic capacity, is GBS strain-specific which also argues for a more tailored strategy of prevention. Given the benefits and risks of IAP, it is important to rethink strategies of preventing GBS disease including alternative strategies such as vaccination and probiotic supplementation. Further studies need to focus on modulating the developing immune-microbiome interplay including the metabolic milieu to the advantage of the vulnerable newborn.

## Introduction

*Group B Streptococci* (*Streptococcus agalactiae*, GBS) are gram-positive, β-hemolytic bacteria that commonly colonize the human intestine, urinary tract and vagina [1, 2] and can be characterized through a variety of molecular properties. The classification of hemolytic streptococci, based on their cell wall C-substance and introduced by Rebecca Lancefield, enables to distinguish different serogroups [3–6]. For GBS, the group-specific structure (group B antigen, group B carbohydrate) consists of a highly branched structure containing rhamnose, galactose, N-acetylglucosamine and glucitol linked by phosphodiester bonds [7]. Further characterization of GBS can be performed by serotyping the sialic acid-rich capsular polysaccharide into one of the ten serotypes Ia, Ib, II-IX [8, 9]. Globally, the most frequently detected serotypes in neonatal GBS infection are III, Ia and V [10]. Based on Multilocus Sequence Typing of conserved housekeeping genes, the sequence type of GBS isolates can be determined [11, 12]. Interestingly, the serotypes are not restricted to specific sequence types [12], still, GBS allocated to sequence type 17 are highly related to serotype III [11, 12].

The critical clinical problem arises from neonatal GBS infections, mainly sepsis and meningitis, which significantly contribute to neonatal death and long-term morbidity. There is an important gap in knowledge regarding the pathways from GBS transmission to colonization and invasive infection, including bacterial and host factors, with the primary objective to find new targets for prevention.

## Colonization and neonatal infection

GBS colonization of pregnant women is a frequent phenomenon. A large meta-analysis covering nearly 300 000 pregnant women worldwide estimated that 18% are colonized with GBS in the rectovaginal tract with remarkable regional and ethnical variation (11%–35%) [13]. GBS cause urinary tract infections, bacteremia, chorioamnionitis, preterm or stillbirth and post-partum endometritis in relation to pregnancy. [1, 14]. In non-pregnant adults, mostly the elderly are affected with urinary tract infections, soft-tissue infections and others.[15–17].

In general, invasive infections during the first three months after birth differ according to host factors, e.g. preterm versus term infants, infection pathways, day of life and pathogenic spectra. Early onset sepsis (EOS) occurs in the first 72 h after birth [18, 19]; some definitions even include a time period of seven days [10, 20]. Causative pathogens in EOS originate from the maternal rectovaginal flora, such as GBS or *E. coli* [21–24], acquired during pregnancy or birth [25, 26]. In preterm infants *E. coli* is a more frequent EOS pathogen than GBS due to the high likelihood of prolonged rupture of membranes and amniotic infection syndrome as underlying cause of preterm birth. The preterm immature lung is regarded as an important site of entry for bacteria causing EOS. In term infants, however, GBS is about 5 times more prevalent as EOS pathogen than *E.coli* [27]. The pathogenesis of EOS involves ascension of GBS from the vagina leading to colonization of the placental membranes and amniotic fluid, potentially provoking chorioamnionitis and/or perinatal transmission during vaginal birth [28]. Most commonly, neonates with EOS or early onset meningitis become clinically apparent within 24 h after birth [29]. [10]. Non-specific symptoms of *EOS* include tachypnea, apnea, grunting, reduced capillary refilling, tachycardia, hypotension, irritability, fever, bulging fontanel, hypothermia and poor feeding [29]. The prevalence of GBS EOS is about 2–7/1000 in preterm infants with a birth weight < 1500 g and about 0.3/1000 in term infants [21, 27].

Late onset sepsis (LOS) occurs between the age of 72 h and three months [18, 30, 31]. In preterm infants Coagulase-negative *Staphylococci, Staphylococcus aureus, GBS, E. coli, Klebsiella* and *Enterobacter* spp. account for 95% of isolates in culture-proven sepsis [24, 32, 33] and are mainly associated with healthcare and invasive measures. In a previous observational study of the German Neonatal Network including preterm infants < 32 weeks of gestation, a GBS-LOS incidence of about 3/1000 was documented [33]. Predominant pathogens of LOS in term infants are GBS, *Staphylococcus aureus* and *E. coli* [24, 32, 33]*.* Key risk factors of LOS in term infants include intensive care or community exposures (e.g., household or skin/umbilical infections) [33]. The GBS LOS rate in term infants is about 0.2–0.3/1000 live births [34, 35]. Transmission routes in GBS LOS cases are not fully understood and may include different body sites such as gut, skin and lung. GBS colonization does not only happen during birth but continues for weeks after birth [30]. In 96% of GBS colonized mother-infant pairs, the GBS serotype is identical. [30]. Prematurity and maternal GBS colonization [36], breast milk feeding and a sibling suffering from LOS in multiples [37] may contribute to postnatal colonization. Even though the general risk of recurrence is low, very low birth weight and a short course of antibiotics (< 10 d) are associated with an increased risk for recurrent LOS [37, 38]. Of note, meningitis more often co-occurs in LOS than in EOS (31 vs. 9%) [10, 39].

The global burden of GBS infections is reflected by data from 2020 which estimated 231 800 EOS and 162 200 LOS cases caused by GBS. Nearly 100 000 infants died and 37 100 survivors developed moderate to severe neurodevelopmental impairment [20]. The case fatality rate of invasive GBS disease in the first three months of life may range between 5–19%, with association to socioeconomic status [10]. Preterm infants are at approximately tenfold higher risk for GBS-related death than term infants (for the United States 23.1–29% in preterm vs. 2.5% in term infants) [21, 22] which seems related to the vulnerability of developing organ systems and the immunological specificities of preterm infants. This points to the urgent need for better prevention strategies and a critical discussion of currently available measures such as intrapartum antibiotic prophylaxis (IAP) in pregnant women at risk for vertical GBS transmission.

## Detection and protection: screening and intrapartum antibiotic prophylaxis

In 1996, consensus guidelines were issued by the American Academy of Pediatrics, the American College of Obstetricians & Gynecologists and the Centers for Disease Control and Prevention recommending IAP in case of a positive GBS-screening or the presence of risk factors [40–42]. In the subsequent years, the incidence of EOS was reduced from 1.8/1000 live births in the early 1990 s to 0.26/1000 live births in 2010 in the United States [43]. Others have shown that if IAP is applied to GBS-colonized women, the risk of early onset sepsis with GBS is around 1% [44]. However, the evidence for the effect of IAP is limited, given the fact that randomized trials are missing.

Internationally, screening-based or risk-based IAP strategies are used. Since the positive predictive value of GBS screening increases with increasing gestational age [45], the American College of Obstetricians & Gynecologists recommends a universal screening of all pregnant women between 36 and 37 weeks and 6 days of gestation with a cultural swab, that is inserted first into the lower vagina and then into the rectum [46].

Risk-based strategies account for factors such as GBS-bacteriuria, premature rupture of membranes (> 18 h before labor), premature labor, maternal fever and a previous child with GBS-infection [47]. Overall, antibiotics are administered with similar frequency in screening-based and risk-based IAP-strategies [48, 49]. Meta-analyses have suggested that the screening-based strategy might be associated with lower rates of EOS than the risk-based strategy [48, 49]. In association with a change from a risk-based towards a screening-based IAP-policy in Finland in 2012, the incidence of early onset GBS disease halved compared to other Scandinavian countries with a risk-based strategy, where the incidence of early onset GBS disease fell by only 11% in the same period [50]. The GBS3 trial, which compared screening versus risk-based approach in a cluster-randomized fashion in 71 maternity hospitals in the UK, completed recruitment in 2024 [51]. The primary outcome include early onset sepsis incidence and cost-effectiveness analysis [51]. First results on the good acceptability of GBS screening and the high rate of results (87%) available ≥ 4 h before birth have been recently published [52]. As either one strategy has yet been demonstrated to be clearly superior, further research is necessary.

Rectovaginal GBS colonization, although mostly stable, can occur transiently [53, 54]: Among 565 GBS- positive women at 35–37 weeks of gestation, only 60% were positive at delivery resulting in a potential overuse of IAP [55]. Conversely, a proportion of GBS-negative women at antenatal testing are tested positive for GBS at the time of delivery and therefore miss the potentially live saving effect of IAP for their newborn [56, 57].

Alternative screening strategies are based on GBS-PCR and may increase predictive test parameters enabling real-time detection ante- or intrapartum under the limitation of assay performance yielding inconsistent results among different assays and no availability of antibiotic resistance [56, 58]. In cases of premature delivery before the timing of antenatal screening, point-of-care testing could enable IAP for premature infants of yet unknown maternal GBS status.

As a consequence of positive screening results, IAP with penicillin and ampicillin are mainly applied worldwide [47]. Concerns regarding IAP include not only potential adverse reactions but also the development of antimicrobial resistance. While penicillin resistance remains rare, clindamycin resistant GBS strains are increasing [22, 59, 60]. Tetracycline resistance is a feature of many GBS strains [59, 61]. This is most likely due to the widespread use of tetracyclines from 1948 onwards, which caused selective pressure possibly leading to an expansion of hypervirulent CC17 clones mediating GBS disease in neonates from the 1960 s [61]. A Taiwanese study analyzed GBS strains before and after implementation of IAP and found an expansion of CC17 strains among invasive GBS isolates (early onset, late onset, very late onset (> 90 days of life)), with no change in antibiotic susceptibility, and likewise an increase in late onset sepsis [62]. Future studies with greater cohort numbers will need to verify whether IAP promotes selection of GBS sequence type17 and whether prevention strategies can be tailored to the context of high GBS-ST-17 transmission risk. Given that the incidence of late onset GBS sepsis is not reduced by the administration of IAP [43], it is important to discuss the pro/cons of IAP including the impact on the delicate immune–microbiome interactions early in life [63].

## Bacterial pathogenicity factors in the context of IAP

The pathophysiology of GBS sepsis includes bacterial factors that are linked to colonization of mucocutaneous surfaces, adhesion, translocation across barriers such as intestinal epithelium and immune evasion [64]. In concert with host susceptibility patterns, the bacterial factors may provide future targets for tailored IAP strategies.

### Arriving and settling in—adhesion and colonization

GBS pilus islands (PI-1, PI-2a, PI-2b) encode long, covalently linked surface structures that mediate adhesion to epithelial and endothelial cells, promote biofilm formation, and can contribute to immune evasion. These functions support vaginal and intestinal colonization of mothers and infants and can facilitate tissue penetration and bloodstream invasion, thus constituting pathogenicity factors [65].

The C5a peptidase ScpB degrades the complement chemoattractant C5a and binds to fibronectin, facilitating epithelial invasion. Its prevalence is higher in strains isolated from newborns with sepsis than in colonizing strains, supporting functional relevance. Other surface adhesins, such as laminin-binding proteins and related LPXTG (sortase recognition motif with leucine (L), proline (P), any amino acid (x), threonine (T), and glycine (G))-anchored molecules (e.g. BibA in non- sequence type 17 strains), contribute to adherence to epithelial/endothelial cells and survival in blood, supporting dissemination in neonates [66, 67].

In a murine model of GBS-colonization, macrophages residing in the lamina propria of the small intestine developed a strong MyD88-dependent inflammatory reaction limiting GBS colonization and dissemination, whereas the transcriptional response of colon macrophages remained largely unaffected [68].

### Getting on track—barrier disruption

Neonates are particularly vulnerable to invasive GBS disease due to intestinal immaturity characterized by leakiness of the gut vascular barrier and immature polarization of cell–cell epithelial junctions, promoting paracellular translocation of GBS. Furthermore, the neonatal gut microbiome is distinct either in abundances and diversity from the adult’s, providing less colonization resistance to GBS [69]. Both factors favor GBS translocation towards invasive disease [69]. It is the specific feature of GBS belonging to CC17 to cross the intestinal barrier in Peyer’s patches by transcytosis through microfold cells (M cells), a process which is dependent on the CC17-specific surface protein Srr2 [70]. Another mechanism of GBS for translocating through the intestinal epithelial barrier is paracellular invasion [71].

The pore-forming toxin β-hemolysin/cytolysin, an ornithine rhamnose-polyene, of GBS contributed to bacterial dissemination in a murine LOS model through transcriptional changes in colonic epithelial cells, which affected intestinal homeostasis, barrier function and immune signaling [72]. In line with that, the metabolic milieu such as maternal diet seems to be important [73]. In vitro experiments with human epithelial cell lines and enteroids suggest that butyrate reduces GBS-induced cell death, GBS invasion, monolayer permeability, and bacterial translocation in vitro, while maternal butyrate intake decreases GBS intestinal burden in offspring mice [73].

The GBS adhesin (HvgA) has been shown to be involved in GBS meningitis development by mediating the translocation of bacteria across choroid plexus epithelial cells and microvascular endothelial cells as part of the blood–brain-barrier [51]. Very recent evidence suggest a model in which monocytes in the dura, recruited from skull and long bone marrow and distinct progenitors depending on disease-inherent needs, are intertwined with the GBS meningitis immunopathology [74].

Although vaginal exfoliation is a protective mechanism against colonization of pathogenic bacteria [75], GBS induce this loss of barrier function with increased transepithelial permeability to increase dissemination and promote ascending infection through integrin and β-catenin signaling pathways [76].

### How GBS outsmarts the neonatal immune system

The interaction between bacterial factors and the developing immune system is considered to be crucial for the successful integration of GBS into microbial ecosystems of the newborn. Neonatal defense mechanisms against GBS primarily rely on innate immunity which may be escaped by GBS through different mechanisms of interaction via (i) capsular polysaccharides, (ii) glycolipids and (iii) hyaluronidases.

First, capsular polysaccharide of GBS contains sialic acids that use inhibitory Sia-binding immunoglobulin-like lectins (Siglecs) on leukocytes to evade host innate immunity through molecular mimicry with sialic acids being a component of the glycocalyx of host cells [77].

Second, GBS glycolipids may attenuate host responses by activating danger sensors in host-microbial interactions. Glycolipids such as glucosyl-diacylglycerol (Glc-DAG), diglucosyl-DAG and lysyl-Glc-DAG are part of the cellular membrane of GBS. The enzyme IagB synthesizes Glc-DAG, from which the other two glycolipids can be produced in further steps [78]. Mature GBS lipoproteins activate TLR2, which is especially important for combatting GBS in an early stage of sepsis in mice [79]. Intriguingly, GBS triggered IL-10 release through TLR2 in murine pups, which was a critical step in the development of neonatal GBS sepsis through inefficient recruitment of neutrophils and therefore impaired pathogen clearance [80]. IagB-deficient GBS show an increased susceptibility to neutrophil killing and antimicrobial peptides in mice with a higher survival after infection and a reduced capacity to persist in tissues and the bloodstream [78].

Third, hyaluronan is usually released from the extracellular matrix by sterile or microbial tissue damage and degraded by host-hyaluronidases to hyaluronan fragments, which induce a proinflammatory immune response in a TLR2/4 dependent manner. GBS-hyaluronidases degrade hyaluronan to disaccharides without proinflammatory activation and thus enable immune evasion and pathogen survival [81].

In addition, several recognition pathways are required for proper defense of innate immune cells against GBS. For example, human macrophages and monocytes recognize GBS via single-stranded RNA and induce a subsequent TNF-α and IL-6 release [82]. In mice, single-stranded RNA sensing is dependent on Toll-like receptors 7 and 13 and the adaptors MyD88 and UNC-938 [82]. If MyD88- and IRAK4 is deficient, patients show a great vulnerability for bacterial infections early in life [83, 84]. Tissue and bone-marrow derived macrophages rely on endosomal TLR13-dependent GBS detection to develop a proinflammatory response to GBS in mice [85], while TLR8 has been identified as essential for GBS sensing in human monocyte-derived macrophages followed by effective antimicrobial production of IFNβ and IL-12 [86]. Upon GBS challenge, neonatal monocyte-derived macrophages obtained from cord blood release more TH1- (IL-12p70) and TH17- related (IL-1β, IL-6, IL-23) and anti-inflammatory (IL-10) cytokines than in adults in vitro, which possibly refers, according to Ravi et al., to a dysregulated immune response to GBS in newborns [87]. Finally, GBS virulence factors such as β-hemolysin trigger caspase 1 through activation of the NLRP3 inflammasome with consequent IL-1β production in bone-marrow derived conventional dendritic cells [88], macrophages [89] and neutrophils [90] in mice.

Intriguingly, GBS characterized by sequence type 17/serotype III is regarded as “hypervirulent” and significantly associated with meningitis through specific pathogenicity factors that enable neurotropism [11, 91]. Translocation of the blood–brain barrier by GBS sequence type-17 is mainly attributed to GBS adhesin (HvgA) [91].

## Immune cell metabolism and defense against GBS

Metabolism is a fundamental determinant of immune cell fate and function, with specific metabolic states actively shaping immune cell activation, differentiation, survival, and effector responses rather than merely supplying passive energy. Several conceptual examples show how metabolic programs in the mother and the infant may affect GBS-related outcomes in the offspring and therefore provide different points of leverage for prevention beyond IAP.

Neonatal defense against GBS can be framed as a metabolically constrained innate response in which key myeloid populations fail to fully engage “adult-like” glycolytic reprogramming and effector programs, with invasive pathogens either failing to trigger or potentially actively subverting these switches [92]. In general, upon sensing bacteria, adult macrophages and dendritic cells undergo a switch from oxidative phosphorylation to aerobic glycolysis, with Tricarboxylic Acid cycle rewiring and increased mitochondrial reactive oxygen species, supporting rapid cytokine production and antimicrobial effector functions [93]. This “Warburg-like” shift is tightly linked to classically activated macrophage polarization, HIF-1α activation, and production of IL-1β, TNF, and other proinflammatory mediators that are critical for control of invasive infections. Neonatal monocytes, on the other hand, show a distinct basal metabolic program with limited glycolytic capacity and impaired ability to upregulate metabolism-dependent innate response genes in a gestational age–dependent manner [92]. Stable maintenance of oxidative phosphorylation may be required for myeloid development in infants, at the cost of emergency responsiveness towards invasive pathogens [94]. Along with these developmentally defined metabolic ranges of glycolytic capacity, GBS displays strain-specific differences in the activation of glycolysis in macrophages [95]. While the molecular basis for these different activation patterns is not yet resolved, these findings support the function of the glycolytic switch as a central bottleneck in neonatal defense against invasive bacterial infections.

Lipid metabolism impacts immunity on multiple levels, with complex interplay between fatty acids, obesity and inflammation. Obesity predisposes for GBS colonization in women [96] and leads to increasing levels of circulating palmitate compared to oleate, which, together with GBS, synergistically amplifies inflammatory responses in decidua, chorion, and amnion cells, with palmitate pre-exposure increasing cytokine output and barrier damage during infection [97].

## Effects of IAP on the infant gut microbiome and health outcome

Microbiota, microbes inhabiting a given ecosystem [98], play a vital role in health and disease throughout life. Functions of the intestinal microbiome include the breakdown of indigestible nutrient components such as human milk oligosaccharides [99, 100], protection against the overgrowth of pathogens [101, 102], biosynthesis of vitamins and short-chain fatty acids [101, 103], regulation of intestinal permeability [104, 105] and interaction with the immune system [106–108]. In addition to antibiotic therapy, birth mode [109–111] and diet [112, 113] are important factors influencing the infant microbiome.

**Fig. 1:**
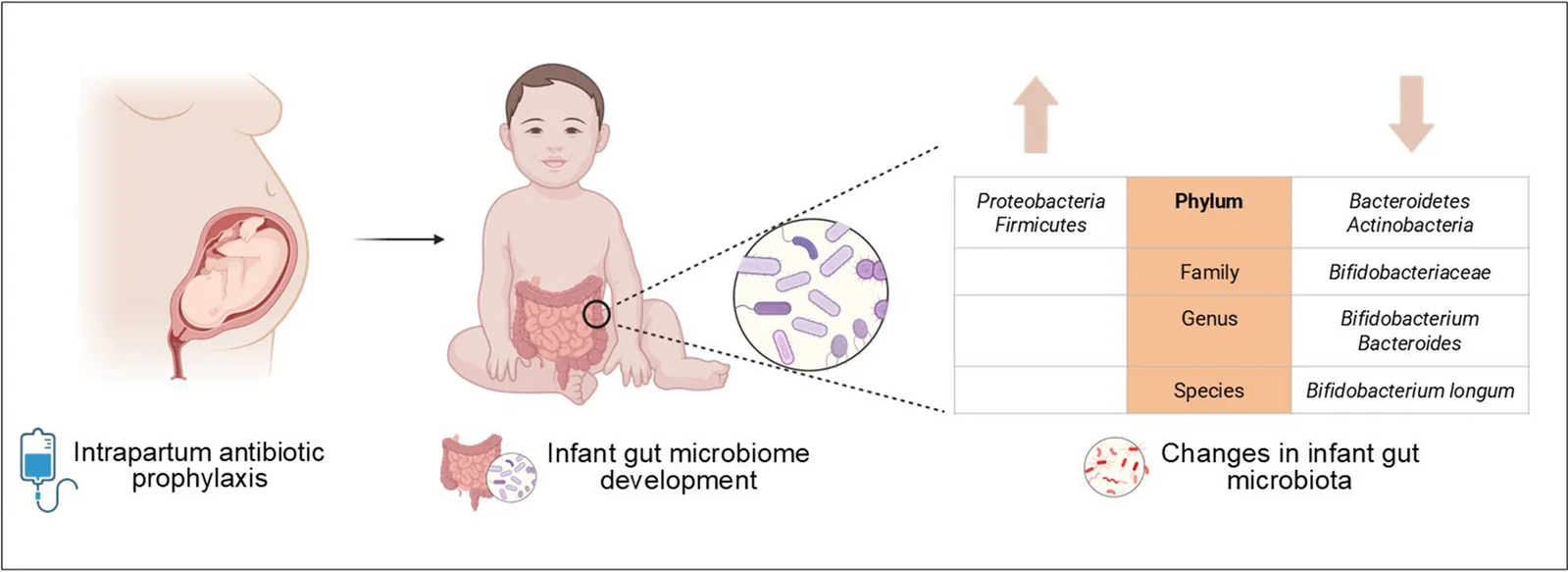
Changes of gut microbiome in IAP-exposed infants during the first tear of age. Created in BioRender. T, J. (2026) https://BioRender.com/bmdd4zc

Considerable effort has been made to evaluate the effects of IAP on the infant gut microbiome [63, 109, 114–137], reviewed in part [138–142], as outlined in Fig. 1. Comparability is limited due to different methodologies (culture-based analyses, 16S rRNA, metagenomics), varying antibiotics for different indications (preterm premature rupture of membranes, caesarean section, maternal GBS colonization), gestational groups and mode of delivery (Table 1). Samples were mainly obtained during the first postnatal weeks, whereas some studies included follow-up periods over the entire first year of age. Additional disruptive factors are postpartum antibiotics, probiotics and different feeding habits (formula, breastfeeding, combination).

**Table 1:** Literature regarding effects of IAP on the neonatal gut microbiome in exclusively vaginally delivered term infants. Studies with only microbial culturing as the diagnostic tool to detect microbial changes were excluded. Symbols: ↔ = unchanged. ▼ = less. ▲ = more. Only significant changes are reported. Abbreviations: PROM—pre-labour rupture of membranes, GBS – Group B streptococcus. W = weeks. M = month. D = days

Author, year	Study population	IAP	Analysis	Sampling	Alpha-Diversity	Phylum level	Familiy level	Genus level	Species level
Aionen et al***.***, [110]	Prospective cohort study (vaginally delivered term infants) - *n* = 27 controls - *n* = 27 IAP-exposed - *n* = 24 postnatal antibiotics (< 24 h after birth until max. 7 d) - *n* = 24 IAP + postnatal antibiotics results reported just for IAP exposed only infants here	IAP: 93% penicillin	16S rRNA, specific region not specified	Infant stool samples at 12 month	↔	IAP exposed ▼*Bacteroidetes*		IAP exposed ▼*Bacteroides*	
Aloisio et al***.***, [112]	Prospective cohort study of breastfed only vaginally delivered term infants - *n* = 10 control - *n* = 10 IAP	IAP ampicillin for GBS positive mothers	16S rRNA V4	Infant stool samples from day 6 or 7	▼Chao1 and Shannon-Index	▼*Actinobacteria, Bacteriodetes* *▲Proteobacteria*		▼ *Bifidobacterium*	
Aloisio et al***.***, [111]	Prospective cohort study of breastfed only vaginally delivered term infants - *n* = 26 IAP-exposed - *n* = 26 controls	IAP ampicillin for GBS positive mothers	RT PCR Lactobacillus spp., Bifidobacterium spp., Bacteroides fragilis, Clostridium difficile, Escherichia coli	Infant stool samples from day 6 or 7				▼ *Bifidobacterium*	
Azad et al***.***, [115]	Prospective cohort study, term infants - *n* = 113 vaginally delivered controls - *n* = 42 vaginally delivered, IAP-exposed (76% in GBS positive mothers, 24% PROM) other caesarean section subgroups	penicillin for GBS positive mothers, antibiotics for PROM not specified	16S rRNA V4	infant stool samples at 3 months, 12 months	Vaginally delivered,. IAP exposed at 3 M ▼Chao 1	Vaginally delivered,. IAP at 3 M ▼*Bacteroidetes*	Vaginally delivered,. IAP exposed at 3 M ▼*Bacteroidaceae* *▲Clostridiaceae* Vaginally delivered,. IAP at 12 M ▲* Clostridiaceae*	Vaginally delivered,. IAP exposed at 3 M ▼*Bacteroides, Parabacteroides, Dorea, Ruminococcus, Sutterella, Collinsella, Prevotella* *▲Clostridium, Enterococcus, Rothia* Vaginally delivered,. IAP at 12 month ▼*Collinsella, Acinetobacter* *▲Clostridia unclassified, Veillonella* (all genera after correction for multiple testing no more significant)	
Chen et al***.***, [117]	Prospective cohort study Term infants - *n* = 375 vaginally delivered, IAP exposed infants - *n* = 876 vaginally delivered control infants Other groups delivered via ceasarean section	penicillin for vaginal delivery with maternal GBS colonization	V4 16S rRNA, qPCR Bifidobacterium und Bifidobacterium longum subsp. infantis	Infant stool samples at 3 month	▼observed OTU, Chao1 index, Shannon ‘s diversity index			▼*Bifidobacterium* (qPCR, 16SrRNA) when stratified for breastfeeding/formula: only significant for at least partially breastfed infants (qPCR, 16SrRNA)	
Coker et al***.***, [118]	Prospective cohort study, vaginaly delivered term infants - *n* = 179 controls - *n* = 55 with IAP: penicillin-like - Other antibiotic classes for IAP	penicillin- like Other antibiotic classes (results not reported)	16S rRNS Sequencing V4,5 Metagenomics subset	Infant stool samples at 6 weeks and one year	penicillin-like ↔			6 W penicillin-like ▼*Bifidobacterium* *Bacteroides* 12 M penicillin-like ▼*Bacteroides* *Clostridium, Megamonas, Streptococcus* *▲Lactococcus, Coprococcus*	6 W penicillin-like ▼*Bacteroides ovatus* 12 M penicillin *▼ Bacteroides fragilis, Eggerthella lenta* *▲Clostridium citroniae, Veillonella dispar, Ruminococcus gnavus*
Corvaglia et al***.,*** [119]	Vaginally delivered term infants - *n* = 35 IAP exposed - *n* = 49 controls	ampicillin	RT-PCR (Lactobacillus spp. Bifidobacterium spp. and Bacteroides fragilis	Infant stool samples at 7 and 30 days				7d ▼ *Bifidobacterium* Feeding type associated with Bifidobacterium count (R^2^(IAP) = 0,22, R^2^(feeding type) = 0,30	
Imoto et al***.***, [120]	Cross sectional study, vaginally delivered term infants - *n* = 4 IAP for GBS - *n* = 6 PROM - *n* = 14 control infants Other groups including infants born via cesarean section	GBS/PROM: ampicillin	16S rRNA V4	Infant stool samples at 1 month (median age 44 d, IQR 41–53)				▼1 M *Bifidobacterium*	
Jokela et al***.***, [122]	Prospective cohort study Vaginally delivered term infants - *n* = 58 control infants - *n* = 25 IAP (penicillin) exposed, Other antibiotic classes and infants delivered via cesarean section (results not reported)	penicillin Other antibiotic classes (results not reported)	16S rRNA and qPCR	Infant stool samples at 1 d, 2 d, 1 W, 4 W, 6 W, 12 W, 6 M, 9 M, 12 M			**Absolute Abundances (not all reported)** 2d ▲*Ruminococcaceae, Veillonellaceae, Enterobacteriaceae* *▼Coriobacteriaceae* 4W ▼*Bifidobacteriaceae* *▲Stretpococcaceae, Staphylococcaceae, Enterococcaceae* 6W ▲*Christensenellaceae, Bacillaceae* 12W ▲*Enterococcaceae* *▼Ruminococcaceae* 9 M ▼*Bacteroidaceae* 12 M ▲*Bifidobacteriaceae*		
Mazzola et al***.,*** [124]	Vaginally delivered term infants - *n* = 13 IAP, (breastfed: BF-IAP, *n* = 7; mixed fed: MF-IAP, *n* = 6) - control *n* = 13 (breastfed: BF-C, *n* = 7; mixed fed: MF-C, *n* = 6)	ampicillin for GBS	16S rRNA V3/V4 qPCR	Infant stool samples at day 7 and 30	7 d BF-IAP compared to BF-C ▼ Chao1 richness estimate, Simpson’s index, Shannon’s index, nr. Of observed species	7 d BF-IAP compared to BF-C ▼ *Actinobacteria*	7 d BF-IAP compared to BF-C ▲*Enterobacteriaceae* d30 BF-IAP compared to BF-C ▼*Veillonellaceae*	7 d BF-IAP compared to BF-C ▼*Bifidobacterium* Compared to zu BF-C d7 qPCR: BF-IAP compared to BF-C and MF-IAP compared to MF-C ▼*Bifidobacterium* D30 MF-IAP i.Vgl. zu MF-C ▼*Streptococcus*	
Nogacka et al***.***, [125]	prospective cohort study: vaginally delivered term infants - *n* = 18 IAP exposed - *n* = 22 control infants	penicillin for maternal GBS	16 S rRNA V3/V4 Gas Chrmoatography for SCFA PCR Resistance genes	Infant stool samples at day 2, 10, 30, 90		10d ▼*Actinobacteria ▲Firmicutes* 90d ▲*Firmicutes*	2d ▲*Prevotellaceae, Rikenellaceae, Muribaculaceae* 10d ▼*Bifidobacteriaceae* *▲Muribaculaceae* *Clostridiaceae* 30d ▲*Muribaculaceae* 90d ▲*Camphylobacteriaceae, Helicobacteriaceae, Prevotellaceae, Muribaculaceae*		
Qi et al***.***, [133]	Prospective cohort study full term delivery - *N* = 18 IAP exposed - *N* = 59 not IAP exposed Stratification for vaginal birth and cesarean section	IAP not specified	16S rRNA V3/4	Infant stool samples at 3 d, 2 M, 6 M Only at 3 d stratification IAP yes/no	↔			3d ▲*Lachnoclostridium, Acinetobacter, Prevotella* ▼*Lachnospira, Agathobaculum, Lactococcus*	
Shao et al***.***, [105]	Vaginally delivered term infants *n* = 314, among these stratification for IAP yes/no with no group size reported Other groups include infants delivered via cesarean section	IAP not specified	Whole genome shotgun metagenomic analysis	At least one sample at day 4,7 or 21. Follow up at 4–12 month for a subset	▼			neonatal period ▼*Bacteroides*	
Stearns et al***.***, [128]	Prospective cohort study of term infants, at least partially breastfed - *n* = 53 control infants - *n* = 14 vaginally delivered, IAP exposed infants Other group delivered via c-section	IAP in vaginally delivered neonates for maternal GBS: penicillin G	16S rRNA V3	Infant stool samples at day 3, 10, week 6 and week 12	IAP d3, d10, 6 weeks ▼alpha-Diversity (Shannon’s diversity, species richness)			12W ▼*Bifidobacterium* *▲Escherichia* For each hour IAP ▼ 7,2% *Bifidobacterium* at 12W ▲ 2,5% *Clostridium* at 12 W	
Tapiainen et al***.***, [129]	prospective cohort study of vaginally delivered term infants, breastfed in the first week of age at one week of age - *n* = 47 Control - *n* = 44 IAP only (YN), - IAP + postnatal AB (until discharge, YY) *n* = 29 - postnatal AB (NY), *n* = 29 at 6 month of age - *n* = 32 control infants - *n* = 31 IAP only (YN) - *n* = 24 with postnatal antibiotic exposure (NY) - *n* = 24 IAP + postnatal antibiotic exposure (YY) results reported only for IAP exposed infants	YN: 86% penicillin IAP	16S rRNA V4-5	Infant stool samples daily between day 1–7 and at 6 month of age		3d-6 month YN ▼*Bacteroidetes* 3 d YN ▲*Firmicutes*		6 M YN ▲*Clostridium*	
Teuscher et al***.***, [59]	Prospective cohort study, all vaginally delivered terms - *n* = 26 controls - *n* = 22 IAP exposed	penicillin	16 S rRNA V3/V4	Infant stool samples at birth, 1 month, 12 month. Maternal rectal swabs at birth	↔				1 M ▼*Bifidobacterium longum* 12 M ▼ *Bifidobacterium longum*
Turta et al***.***, [130]	Case control study, all infant vaginally delivered terms, 1 month breastfed - *n* = 24 controls - *n* = 24 IAP-exposed	Benzyl-penicillin or cephalosporins	16srRNA V3/4	Infant stool samples at one and six month	▼1 M Chao1 index (richness)		1 M ▼*Erysipelotrichaceae*	1 M ▼*Collinsella* *▲Clostridium* 6 M ▼*Colinsella* *▼Propionibacterium*	

In vaginally delivered term newborns, on phylum-level, a reduction of *Bacteroidetes* in the first month after birth (until one year of age [114, 116, 119, 128, 133]) and *Actinobacteria* (in the first week after birth [115, 128, 129]) has been consistently observed following IAP, whereas *Proteobacteria* (in the first week after birth, [116]) and *Firmicutes* (until 12 month of age [114, 129, 133]) were more abundant. On genus-level, *Bifidobacterium* was less abundant within the first three months *postpartum* [115, 116, 121–124, 128, 132], likewise a reduction in *Bacteroides* has been reported until the age of 12 months [109, 114, 122]. For *Clostridium*, different results have been reported [114, 122, 133, 134]. On species-level, a reduction of *Bifidobacterium longum* has been reported up to one year of age following IAP [63]. Interestingly, Stearns et al*.* reported a 7.2% reduction in the abundance of *Bifidobacterium* at the age of three month for each hour IAP [132]. A meta-analysis has been performed by Grech et al*.* on five studies with fecal sampling between one week and three month reporting a nonsignificant reduction in infant α-diversity following IAP exposure [143].

With regard to health development of children, IAP was associated with increased childhood BMI or weight gain in the first five years of life in vaginally delivered, IAP-exposed children [144, 145], which corresponded to a higher weight of 240 g to 320 g at the age of five years [144, 145]. Sidell et al. conducted a follow-up even up to the age of ten years and found an increased BMI in vaginally delivered, IAP-exposed infants with a ∆BMI that even increased from 0.09 at the age of five years to 0.14 kg/m^2^ at ten years [146]. By contrast, two smaller studies yielded no significant weight changes after IAP-exposition [147, 148].

Regarding the risk of asthma in vaginally delivered, IAP-exposed infants, there are conflicting results: Ainonen et al*.* found in a large population-based cohort study of 9733 exposed and 35 842 unexposed infants a higher risk during the first five to six years in an analysis adjusted only for child’s sex and year of birth [149], whereas Dhudasia et al*.* did not detect higher occurrence of asthma in a retrospective cohort consisting of 1919 IAP-exposed and 6989 not exposed infants [150]. Possible reasons for the ambiguous results may lie in the heterogeneous pathophysiology of asthma, which involves many contributing factors (e.g. genetic background, environmental influences such as pollutants), and thus in the need for very large, ideally prospective cohort studies with sufficient statistical power and adequate follow-up periods to diagnose infant asthma.

Three studies did not find an association between atopic dermatitis and *intrapartum* antibiotic exposure in vaginally deliveries during infancy [149–151], whereas Hong et al*.* found a significantly higher occurrence of atopic dermatitis among IAP-exposed infants [152]. Hong et al*.* compared IAP-exposed infants of GBS positive mothers to reference infants including those with ≤ 4 h antibiotic exposure or other IAP than for GBS in a retrospective study design putting up with the risk of recall bias. Vaginally delivered infants exposed to IAP were also significantly more often exposed to postnatal antibiotics. Therefore, clear inclusion and exclusion criteria should be defined regarding the research question in order to identify clinically relevant associations.

Dhudasia et al*.* did not find a higher incidence of food allergy among IAP-exposed, vaginally delivered infants [150].

Hutton et al*.* detected a higher incidence of atopy (asthma or reactive airway disease, eczema, allergy) among IAP-exposed, vaginally delivered infants at the age of one year compared to not exposed infants [147].

In a Finnish prospective population-based cohort study, *intrapartum* antibiotic exposure was associated with the occurrence of autoimmune diseases (such as type 1 diabetes mellitus, celiac disease or rheumatoid diseases) among 9733 IAP-exposed children and 35 842 children without IAP during a follow-up period of five to six years [149].

Both microbiome alterations and health-related outcomes such as obesity, associated with IAP in vaginally delivered infants, suggest an immune imprinting due to alterations in microbiome colonization of the newborn with possible health implications throughout infancy. As up to half of delivering women receive IAP in the US [153], the possible long-term effects of the IAP affect many and are associated with considerable costs in the healthcare system. Therefore, future studies to ensure microbiome-associated health alterations after IAP-exposure are warranted to detect mechanistic relationships. Additionally, alternative strategies to prevent early onset GBS disease should be considered.

## Alternative strategies to IAP

Alternative strategies for the prevention of early onset GBS disease to IAP are currently under development. The World Health Organization has determined the development of a safe, effective and affordable GBS vaccine for maternal immunization during pregnancy as a strategic goal to prevent GBS-related stillbirth and invasive GBS disease in neonates and young infants, applicable in high-, middle and low-income countries [154]. The GBS-vaccine should preferably include a one dose regimen [154].

The concept of diaplacental transfer of immunoglobulins particularly in the last trimester [155, 156] is already being utilized for vaccinations against pertussis [157, 158], SARS-CoV-2 [159], influenza [157], and tetanus [160]. Interestingly, in infants born to GBS colonized mothers, the half-life of anti-GBS-capsular polysaccharide-IgG is 27.4 days and not influenced by factors such as maternal age, ethnicity and infant sex, notably in a relatively small cohort [161]. Since the serotypes Ia, Ib, II, III and V account for 97% of invasive GBS isolates of infections during the first three months [10], a pentavalent GBS vaccine can reduce the number of stillbirths and premature births [86] that cannot be prevented by IAP. Taking into account possible teratogenicity versus a preferably early timepoint to prevent prematurity, vaccination in the early third trimester could be appropriate [162]. As of yet, the protective effect of pregnancy vaccinations for the offspring cannot be simply deduced from experience with vaccinations against respiratory infections such as pertussis.

Currently, capsular polysaccharide vaccines (monovalent or multivalent: bi-, tri-, tetra- or hexavalent) and a surface subunit protein vaccine are under development undergoing Phase-1 or Phase-2 trials [160, 163–183]. GBS conjugate vaccines induce better immunological responses than non-conjugated GBS vaccines [162]. For conjugated vaccines, diphtheria or tetanus toxoid or CRM197, a non-toxic variant of diphtheria toxin, serve as carrier proteins [184–186]. Pfizer started the recruitment for a Phase-3-trial to evaluate a hexavalent GBS-vaccine in 6000 healthy pregnant women and their neonates in August 2025, primary completion is estimated for November 2028 [187].

A systematic review of GBS-vaccines in development conducted by Bjerkhaug et al*.* in 2024 included 5765 participants, among these 1325 pregnant women [184]. Bjerkhaug et al. ascertained a peak in GBS-antibodies 4–8 weeks after vaccination remaining elevated for 6–12 months. The placental transfer ratio (ratio of maternal GBS-antibodies to infant or cord blood GBS-antibodies) ranged between 0.4–1.4, indicating, according to Bjerkhaug et al*.,* that GBS-antibodies cross the placenta and can protect infants from invasive GBS disease [184].

However, the duration of postnatal protection by pregnancy vaccination remains unclear. With respect to influenza vaccination in pregnancy, protection wanes at 4 postnatal months [188].

Interestingly, one study determined a half-life of GBS-antibodies in infants of 42 days [173], whereas another study measured an infant GBS-antibody level of ≈25% three months after birth [172]. In a previous study, sera were prospectively collected from 473 GBS non-colonized and 984 colonized pregnant women who delivered healthy neonates and from 153 mothers of infants with GBS disease. Antibody levels against capsular polysaccharides and pilus proteins were significantly higher in GBS colonized women delivering healthy babies than in mothers of neonates with GBS disease [189]. Remaining questions relate to the impaired transplacental transfer in preterm infants, and the potential emergence of very late GBS sepsis with waning antibody levels in the face of persisting GBS colonization pressure.

Bjerkhaug et al*.* also analyzed adverse events and concluded that even though mild reactogenicity symptoms such as pain at the injection site, tenderness or local swelling as well as fatigue and headache occurred, no differences in serious adverse events were observed between vaccinated and placebo groups, especially no deaths were reported [184]. As none of the capsular polysaccharide vaccines under development cover all serotypes, the risk of capsular switching during pregnancy remains and GBS of not vaccinated serotypes could emerge [185]. Here, protein-based vaccines offer a serotype-independent protection against GBS targeting the N-terminal domain of the alpha-like surface protein family [186] or the pilus antigen [190].

In a murine model, sustained GI colonization following both perinatal and postnatal exposure to GBS resulted in late-onset sepsis in 21% and 27%, respectively. Intestinal colonization with GBS induced an endogenous GBS-specific IgG response within 20 days of exposure. Although maternal vaccination with whole-cell GBS induces production of GBS-specific IgG in dams and a transplacentar passage to their offspring, the nest protection did not save from GBS mortality after postnatal exposure [191].

Apart from vaccination, the use of probiotics to reduce maternal GBS colonization and therefore prevent vertical transfer are currently the subject of research. Probiotics are defined as live microorganisms that if administered in appropriate amounts provide a health benefit to the host [192]. Proposed mechanisms for the anti-GBS effects of often applied probiotics for improvement of vaginal health such as *Lactobacillus* spp. or *Streptococcus salivarius* is through acidification by lactic acid and hydrogen peroxide and high cell adhesion to vaginal epithelial cells as well as immunomodulation [193]. A meta-analysis conducted by Menichini et al*.* on five randomized controlled trials ascertained a reduction in rectovaginal GBS colonization of pregnant women at 35–37 weeks of gestation following probiotic supplementation (four studies: *Lactobacillus rhamnosus GR-1* and *Lactobacillus reuteri RC-14L* [194–197], one study: *Lactobacillus jensenii Lbv116, Lactobacillus crispatus Lbv88, Lactobacillus rhamnosus Lbv96* and *Lactobacillus gasseri Lbv159* [198]), treatment after 30 weeks of gestation was associated with a more pointed reduction [199].

Hanson et al*.* conducted a meta-analysis with three randomized controlled trial, two prospective cohort studies and one *quasi* experiment [194, 195, 197, 200–202] on the effects of probiotics on rectovaginal GBS colonization including 709 participants and found a significant reduction of GBS colonization after probiotic administration and, most importantly, no adverse events were reported [193].

Probiotic administration of a probiotic mixture containing *Enterococcus faecium* L3, which has anti-GBS capacities due to bacteriocins, *Bifidobacterium animalis ssp. lactis*, *Lactobacillus casei* and *Lactococcus lactis ssp. lactis* for twelve weeks (24 to 36 weeks of gestation) in women with recurrent genitourinary infections was associated with a reduced occurrence of premature rupture of membranes, decreased detection of GBS in rectovaginal swabs between 36 and 37 weeks of pregnancy and thus a reduced need for application of IAP [203]. Notably, in this study no randomization was adopted but allocation was based on decision of the patient herself or according to the physician’s recommendation in relation to intestinal symptoms and no blinding of physicians during sampling was reported. Furthermore, no placebo for controls was administered. A trial conducted by Shirani et al*.* did not detect a reduction in GBS colonization following a not further specified probiotic supplementation for 30 days after vaginal GBS sampling at on average 27 weeks of gestation, the control and intervention group consisted of 32 participants each [204].

It should be noted that probiotic studies are heterogeneous in sample size, timing of recruitment, strain, dosage and duration of intervention which may compromise interpretation of findings [185]. In addition, probiotics are inconsistently classified, for example as food, dietary supplement or drug [205], and therefore food safety underlies controls of different rigor. In future studies, different probiotic strains should be evaluated against each other in terms of their anti-GBS efficacy in large studies with adequate study design including a double-blind, placebo control regimen, well-defined bacterial strains and exact timeframes for probiotic supplementation. Both, vaccination and the use of probiotics during pregnancy offer the possibility of preventing not only EOS, but also premature birth and stillbirths.

Future strategies for preventing invasive GBS disease are, for example, postbiotics such as the use of Lactobacillus extracellular vesicles to support microbiome homeostasis and phage therapy [206, 207]. Enzymatic, GBS-specific lysogenic bacteriophages such as PlyGBS hold specific potential for reducing GBS colonization as shown in a mouse model [208] and, consequently, limiting transmission to the neonate. In addition, the alarmin S100A8 may support the defense against GBS infection mainly by recruitment and activation of phagocytes [209] and by chelation of zinc which directly inhibits GBS growth by creating a metal-starved niche [210]. In a clinical context, high amounts of S100A8/A9 levels in human breast milk confer antimicrobial activity to target GBS which provides the basis for clinical studies on nutritional supplementation in neonates with low S100 A8 levels [211].

## Conclusion

In newborns, the immune system is programmed to meet the challenges related to managing the transition from full intrauterine dependence on maternal immunity and metabolism to far reaching independence [212]. The neonatal period is therefore a susceptible time period for infection with GBS. IAP reduces the risk of early-onset GBS sepsis at the potential expense of more LOS cases. Moreover, it is associated with changes of the infant gut microbiome in the first year of age and health-related outcomes such as obesity, both could indicate an immune imprinting through distinct microbial colonization patterns with possible health implications throughout infancy. It is therefore time to rethink GBS prevention including the need for better defined subsets of pregnant women who qualify for IAP and infants at specific risks. Alternative approaches such as vaccination hold promise to prevent stillbirths, premature births and GBS infections but need to be explored in large cohorts. New targets of prevention should consider the pathogenicity factors of GBS and modulating the developing immune-microbiome interplay including the metabolic milieu to the advantage of the vulnerable newborn.

## Data Availability

All data for evidence synthesis will be available upon request to the corresponding author.
